# Laryngeal Hemorrhage

**Published:** 1882-08

**Authors:** Jefferson Bettman

**Affiliations:** Acting Resident Medical Officer to the Throat and Chest Hospital, Golden Square, W., London, etc.


### ARTICLE II.

LARYNGEAL HÆMORRHAGE. By JEFFERSON BETTMAN, M.D., Acting Resident Medical Officer to the Throat and Chest Hospital, Golden Square, W., London, etc.

On account of the meager literature on this subject and the undue importance which could easily be attached to such a symptom, the cause remaining unrecognized, the following case, which lately came under my observation may not be without interest or devoid of some practical bearing.

Mrs. C. B., aged forty-five, a widow, mother of four children, has enjoyed, up to recent date, comparatively good health being troubled only now and then with attacks of rheumatism. She is more or less subject to colds and during the past few winters has suffered from cough with little expectoration. Family history good; parents died at a ripe old age; children healthy; husband was killed at a railroad accident. Her present illness began a week ago, and, according to her statements, seems to have been brought on by exposure to a draught. The following day she experienced a feeling of heat and slight constriction in the throat. Her voice became husky, a short, hacking cough set in. During the course of the next few days these symptoms were aggravated; her throat and chest became very painful, cough very harassing with little or no expectoration. On the eve of the fifth day, during an almost suffocating paroxysm of cough, she suddenly began to expectorate blood, which though at first small in quantity gradually increased in amount and reached, according to her statements, in the aggregate during the same night to more than half a teacupful. She now felt an intolerable burning in the throat, to use her own expression, "as if something were passing down the wrong way." Matters only became worse; she continued to expectorate fluid blood, grew feverish and quite weak.

*Status præsens.* Patient seems poorly nourished and is in a pale, anæmic state. Breathing not perceptibly disturbed, voice is almost aphonic and conversation can only be carried on with visible exertion. Complains of tickling in the throat and a burning pain over the sternum. While interrogating her, patient had a violent attack of coughing, explosive in its type. During the paroxysm she coughed up about half a teaspoonful of blood. I thus had opportunity to study the true character of the expectoration. It consisted, excepting a few dark brown crusts, of pure, fluid blood, bright red in color. Pulse slightly accelerated, temperature normal. After careful examination the thoracic organs were found to be healthy. Rhinoscopic examination both anterior and posterior revealed nothing abnormal. Mucous membrane of mouth and naso-pharynx, pale and glistening. Patient was very nervous and the throat so irritable, that I succeeded only after repeated attempts in getting a satisfactory view of the larynx and trachea—amply sufficient, however, to remove all doubts regarding the cause and true state of affairs. The entire laryngeal mucous membrane and that of the trachea as far as I could see, was uniformly congested, deep red in color and here and there covered by small dark brown crusts. The vocal cords were very hyperæmic, the free borders very much thickened and slightly irregular. Arytenoids and inter-arytenoid fold were involved in the intense congestion. The ventricular bands however presented the interesting and main feature of the case. They were almost livid in color, very much thickened, overlapped in part the vocal cords and completely obliterated the ventricles. They were in part covered with fresh and dried blood. With a good light, the superficial vessels of the congested mucous membrane could be easily seen as dark tortuous lines converging toward two bleeding spots seated symmetrically on the lateral surfaces of the ventricular bands. These spots were indicated by two small coagula, the size of split peas. Epiglottis congested, crest slightly thickened.

*Diagnosis.* Acute laryngo-tracheitis, rupture of small vessels and subsequent hæmorrhage from ventricular bands.

*Tveatment* consisted, locally, in a light application of nitrate of silver (fused on a laryngeal probe) to the bleeding spots. The main treatment, however, was directed toward the "fons et origo mali"—the laryngo-tracheitis and consisted in mild laxatives, inhalations of vapor of benzoin, pellets of ice and a cold water dressing to the neck.

The application of these remedies was followed by great and rapid amelioration of all the symptoms. Two days later I again saw the patient. There was great improvement in her general physique ; cough, though still present, was much less troublesome ; all bloody expectoraticn had ceased. With the aid of the laryngoscope, the cords were found much less injected, the ventricular bands reduced in size and the sites of the former hæmorrhages occupied by two small, white eschars. She rapidly recovered and about a fortnight later had regained her normal voice ; the larynx, barring a slight congestion of the mucous membrane, had taken on its natural appearance.

Laryngeal hæmorrhage, due to direct traumatic influence, is not, an uncommon occurrence and many instances have been recorded. It can be easily understood how foreign bodies inpacted in the larynx or trachea can produce hæmorrhage by lacerating the tissue. Gross* has recorded such a case, where a boy, aged three years, received into his air passages a peice of bone and suffered at imtervals during a period of sixty years, from hæmoptysis, cough, dyspnœa etc. The same condition may arise after wounds or bruises of the larynx and several observations are cited in literature where hæmorrhages were induced by ulcerative processes. In a case of Tuerck's† syphilis of larynx, the bleeding had a fatal termination. The extensive ulceration involving the right sinus pyriformus not only exposed the greater corner of the hyoid bone, but produced erosion of the lingual artery with above mentioned result. Independent of any extraneous causes or ulcerative action, laryngeal hæmorrhage is of rare occurrence. I of course exclude all cases of hæmorrhage into the submucous connective-tissue, occurring occasionally in variola hæmorrhagica (Bogros‡) and scorbutus (Ruehle§) and limit myself entirely to such complications as arise during the course of an acute inflammatory congestion. In the case under discussion the sudden onset of a bloody expectoration in an acute laryngitis is as rare as its individual features and clinical history are interesting.

* " Foreign Bodies in the Air-Passages " p. 172, Phila., 1854.

† " Klinik der Kehlkopfkrankheiten " S. 402.

‡ Vide Sestier, " Traité de l'angine laryngée œdémateuse " pp. 63 and 114. Paris, 1852.

§ Kehlkopfkrankheiten S. 172.

The symmetrical position of the bleeding spots is probably due to the fact, that during the paroxysms of cough, the swollen and congested ventricular bands were tightly pressed against each other, an erosion of epthelium followed by rupture of the turgescent vessels and subsequent hæmorrhage. The trickling of blood into the larynx proved a fresh source of irritation, provoked increased cough ; this again favored great congestion with subsequent hæmorrhage and thus a sort of vicious circle was established.

Semelder* has recorded a very interesting case of laryngeal hæmorrhage setting in after violent emesis. He found a mottled appearance (submucous extravasation) of the vocal cords and a small bloody coagulum seated on the anterior extremity of the right ventricular band. He also attributed the sudden hemorrhage to a rupture of congested vessels, induced by the muscular strain during emesis. Navratil† has described a case under the name "chorditis hæmorrhagica." Here the hæmorrhages took place on the upper surface of the cords and after removing the dried crusts the underlying cord was found red and swollen. Lewin‡ has made a similar observation. Mandl§ has seen a hæmorrhage proceed from the ventricles of Morgagni in the case of an old lady suffering from laryngitis. A few years ago Fraenkel|| published another example of this rare occurrence. He applied the title "Hæmorrhagic Laryngitis" to it, but I fully concur with Dr. Mackenzie¶ "that it is scarcely necessary to give a special name to so rare and accidental a condition." In Fraenkel's case there was also great stridor and dyspnœa and, as he himself adds, in such a case a correct diagnosis could be established without the aid of the laryngoscope.

While in Vienna, another case of laryngeal hæmorrhage came under my notice. The patient had been under the care of Prof. Stoerk for some time. A large papillomatous excrescence had developed on the posterior laryngeal wall, a little below the level of

---

* Laryngoskopie, Wien 1863 S. 33.

† "La'yngol Beiträge" Leipzig, S. 18.

‡ "Inhalations therapic" S. 328.

§ "Maladies du Larynx" p. 644 Paris, 1872.

|| "Berliner Klin. Wochenschrift" No. 2, 1874.

¶ "Diseases of Nose and Throat" vol. I. p. 268.

the arytenoid cartilages. The patient as yet presented no sign of pulmonary complaint, still the appearance of the growth and its more characteristic position, was simply sufficient to establish a diagnosis of incipient phthisis (Stoerk*). I followed the case with interest and the Professor kindly placed him under my personal care. One morning the patient looking pale and anæmic came to the clinic and gave the following account: During the night he had a violent fit of coughing; suddenly he felt the sensation " as if something had given way " in his throat, this being followed immediately by a little gush of blood. I immediately examined him with the laryngoscope and was not a little surprised to find a state of affairs, varying considerably from that existing heretofore. The former round, granular looking excrescence was now cleft longitudinally and on the patient's inspiring deeply I found that the fissure extended through the greater part of its substance. The cords, ventricular bands etc, to all appearances perfectly normal, were here and there covered with blood. This condition, occurring inter-arytenoid, has been lucidly described by Stoerk under the title " Fissura Mucosa."†

The fissure was thoroughly cauterized with argentum and all hæmorrhage ceased. It is further interesting to add that a few months later, physical diagnosis disclosed infiltration of apex of right lung.

These excrescences are generally quite firm in consistence, its German term " Schwielen " being quite expressive, and it can be readily comprehended how, during an attack of coughing, its stiff unyielding substance had suddenly given way to the great muscular strain of the underlying laryngeal muscles.

The quantity of blood expectorated in laryngeal hæmorrhages must necessarily vary according to the gravity of each case. In the one published by Fraenkel, the patient at one time coughed up a cupful of blood. In my case the quantity was much less, still the continuous oozing during three or four days was enough to seriously debilitate her.

These cases are however exceptional and judging from the few observations on record, the prognosis as a rule is favorable as

* " Klinik der Krankheiten des Kehlkopfes " Stuttgart, 1876.

† Virchow's Archiv Bd. lx.

regards life. In the more aggravated forms such as in Fraenkel's and Lewin's cases where there was great stridor and dyspnœa, the gravity of the condition is naturally greatly enhanced. Local treatment must consist in application of styptics, or, in severe cases, of caustics to the bleeding points. Ice is a valuable adjunct in the treatment for it not only assists in constringing the vessels but acts very soothingly in reducing the pain and heat of the parts. As mentioned above, the main treatment must be directed towards the cause of the hæmorrhage; if (as it is in the majority of cases), due to acute congestion or inflammation of the larynx, it should be treated accordingly. In the very grave forms where all other resources have proved of no avail, and the stridor and dyspnœa is so great, as to call for rapid action, surgical interference is no doubt indicated. J. B.

---

I am under great obligations to Dr. Morell Mackenzie, who in this as in former occasions has kindly permitted me to avail myself of his clinical material.

---

### Epilepsy with Mania caused by Masturbation.

The patient, a girl sixteen years of age, had healthy parents. The labia majora and the introitus vaginæ were enlarged. The menses had been regular. She was found on the street in convulsions, the pupils enlarged. At night she would scream at the highest pitch of her voice, and she said that an alligator was approaching her and about to devour her. She was often unconscious for two hours. After that she had maniacal attacks, and tried to kill everybody present. She moved slowly, and voided the urine eight to ten times a day. At last she was sent to an insane asylum. The physician, who watched her closely, discovered that she masturbated, and she confessed that the attacks had come on after each masturbation. She consented to have her hands secured at night, and the nurse watched her closely in the daytime. After three months she was dismissed entirely well, and comparatively cured of the vice of masturbation.

